# The Impact of Dual and Triple Energy Window Scatter Correction on I-123 Postsurgical Thyroid SPECT/CT Imaging Using a Phantom with Small Sizes of Thyroid Remnants

**DOI:** 10.3390/life14010113

**Published:** 2024-01-11

**Authors:** Konstantinos Michael, Savvas Frangos, Ioannis Iakovou, Antonis Lontos, George Demosthenous, Yiannis Parpottas

**Affiliations:** 1 Department of Mechanical Engineering, Frederick University, Nicosia 1036, Cyprus; 2 Department of Medical Physics, Bank of Cyprus Oncology Center, Nicosia 2006, Cyprus; 3 Department of Nuclear Medicine, Bank of Cyprus Oncology Center, Nicosia 2006, Cyprus; 4 School of Medicine, Aristotle University of Thessaloniki, 54621 Thessaloniki, Greece; 5 Frederick Research Center, Nicosia 1036, Cyprus

**Keywords:** thyroid phantom, thyroid remnants, postsurgical diagnostic thyroid imaging, nuclear imaging

## Abstract

I-123 is preferential over I-131 for diagnostic SPECT imaging after a thyroidectomy to determine the presence and size of residual thyroid tissue for radioiodine ablation. Scattering degrades the quality of I-123 SPECT images, primarily due to the penetration of high-energy photons into the main photopeak. The objective of this study was to quantitatively and qualitatively investigate the impact of two widely used window-based scatter correction techniques, the dual energy window (DEW) and triple energy window (TEW) techniques, in I-123 postsurgical SPECT/CT thyroid imaging using an anthropomorphic phantom with small sizes of remnants and anatomically correct surrounding structures. For this purpose, non-scatter-corrected, DEW and TEW scatter-corrected SPECT/CT acquisitions were performed for 0.5–10 mL remnants within a phantom, with 0.5–12.6 MBq administered activities within the remnants, and without and with background-to-remnant activity ratios of 5% and 10%. The decrease in photons, the noise and non-uniformity in the background region due to scatter correction were measured, as well as the signal-to-noise ratio (SNR) and the contrast-to-noise ratio (CNR) from small remnants. The images were also visually evaluated by two experienced nuclear medicine physicians. Scatter correction decreased photons to a higher extent in larger regions than smaller regions. Larger remnants yielded higher SNR and CNR values, particularly at lower background activities. It was found from the quantitative analysis and the qualitative evaluation that TEW scatter correction performed better than DEW scatter correction, particularly at higher background activities, while no significant differences were reported at lower background activities. Scatter correction should be applied in I-123 postsurgical SPECT/CT imaging to improve the image contrast and detectability of small remnants within the background.

## 1. Introduction

In differentiated thyroid cancer, Single-Photon Emission Computed Tomography (SPECT) imaging using I-123 after a thyroidectomy can offer valuable insights regarding the presence and size of residual thyroid tissue or metastases. I-123 is preferential to I-131 in SPECT diagnostic thyroid imaging due to its much shorter half-life and better resulting image quality [1,2,3]. Thus, it facilitates precise restaging of the disease, enabling the application of personalized radioiodine therapy.

When a cyclotron (energy range > 17 MeV) and a PET/CT modality are available, postoperative thyroid imaging with I-124 PET/CT can be performed. This imaging offers a superior image quality due to the absence of physical collimation and a shorter coincidence time window [4]. I-124, with a half-life of 4.2 days compared to I-123 with a half-life of 13.2 h, enables the study of more extended metabolic processes but also increases patient radiation exposure.

Several factors affect the quality of I-123 SPECT images, such as the sensitivity and resolution of the detectors, partial volume effects, attenuation and scattering [5,6,7]. The latter is a main issue since I-123 emits photons of 159 keV (83.4%) and 529 keV (1.3%) [8]. The relatively high-energy 529 keV photons can penetrate the septa of the collimators, backscattering into the SPECT detector and contributing to the main energy window at 159 ± 10 keV [6]. Low-energy collimators with thin septa are utilized in tumor imaging to achieve a high resolution, high count rate and increased contrast. Thus, scatter correction (SC) is imperative [6,9].

A number of techniques are available, and they can be employed to perform SC. However, in clinical practice, scatter window-based techniques are preferred due to their simplicity [10]. In the last 30 years, DEW and TEW scatter correction techniques have been extensively applied in SPECT imaging [10,11,12]. A number of researchers have attempted to assess these techniques for different radionuclides, collimators, SPECT systems and imaging procedures [13,14,15,16,17,18,19].

Most research on the assessment of SC with I-123 has been conducted for cardiac SPECT imaging. In a patient study, Fletcher et al. [20] assessed the cardiac uptake from I-123 MIBG DEW scatter-corrected SPECT images with different collimators, and they concluded that Low-Energy High-Resolution (LEHR) collimators together with SC should be used. In a similar study, Kobayashi et al. [21] utilized the DEW scatter correction method, and they concluded that SC is imperative to standardize cardiac uptake in I-123 MIBG SPECT imaging. In a cardiac phantom SPECT study, Inoue et al. [22] applied TEW SC and reported that it increased the overall image quality. In a more recent study, Papanastasiou et al. [23] also utilized a cardiac phantom and TEW SC. They demonstrated that SC improved the SPECT image quality when used with LEHR collimators compared to uncorrected images. Lagerburg et al. [9] used the NEMA (NU2-2007) phantom to compare the effectiveness of different window-based scatter correction methods on SPECT imaging in terms of the I-123 intensity ratios between spheres and background cavities. These ratios did not differ significantly from the real ratios when using the LEHR collimators together with TEW or DEW SC methods and when considering different sizes of spheres.

SPECT brain studies with I-123 also investigated the performance of SC. Hayasi et al. [24] utilized TEW SC in a patient study, and they have shown that SC provided more accurate cerebral blood flow measurements than no scatter correction. In a phantom study with I-123 in brain-like cavities and in the background, Yang et al. [25] employed DEW SC and concluded that the image contrast and visual image quality were improved compared to non-scatter corrected images.

However, a direct comparison of DEW and TEW SC on I-123 SPECT imaging utilizing small sizes of volumes surrounded with appropriate anatomical scattering structures has not been performed yet. For this purpose, an anthropomorphic neck–thyroid phantom was used, specifically designed for postsurgical thyroid SPECT imaging, which enclosed anatomically small sizes of thyroid remnants as well as other tissue-equivalent human-shaped structures. The impact of DEW and TEW SC on I-123 SPECT/CT thyroid images was quantitatively and qualitatively evaluated for various administered activities, small sizes of thyroid remnants, and background-to-remnant activity ratios.

## 2. Materials and Methods

**Figure 1:**
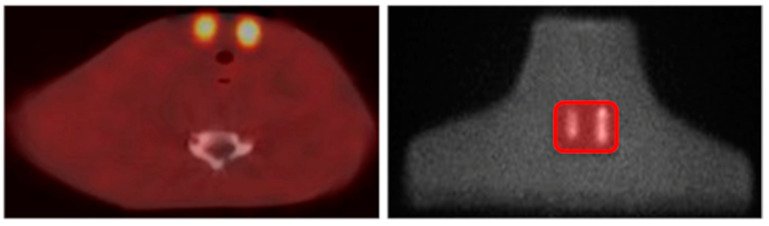
(**Left**) Fused SPECT/CT axial slice through the centre of the 1.5 (left side of the image) and 3 mL (right side of the image) remnants. The lateral direction is defined from left to right and the anterior-posterior (AP) direction from top to bottom. (**Right**) Planar AP image of the phantom with an administered I-123 activity of 0.37 MBq/mL within the remnants and a background-to-remnant activity ratio of 5%. The red frame shows the section with remnants [26].

I-123 SPECT/CT images from a neck–thyroid phantom with thyroid remnants were utilized. The phantom was specifically designed for postsurgical thyroid imaging and was developed using 3D printing and moulding techniques [26]. The phantom can accommodate remnants of various small sizes, at clinically relevant positions, and can simulate different background-to-remnant activity ratios. Other structures such as the trachea, oesophagus, cervical spine and clavicle were also anatomically enclosed within the phantom. For this study, remnants of 0.5, 1, 1.5, 3 and 10 mL were used. Figure 1 shows a fused SPECT/CT axial slice and a planar anterior-posterior (AP) image of the phantom where I-123 activity was administered within the 1.5 and 3 mL remnants and the background region.

All images were acquired using the dual-head GE Infinia Hawkeye 4 SPECT/4-slice-CT hybrid scanner (GE Healthcare, Milwaukee, WI, USA) at the Bank of Cyprus Oncology Centre following the clinical protocols. All acquisitions were performed using the LEHR collimators in 180° (H mode) orientation, with 60 projections, 35 s per projection and over 180° of rotation, thus covering an angular range of 360°. The matrix size was 128 × 128. Autocontouring was enabled and the images had an isotropic pixel size of 4.42 mm. A Butterworth filter (cutoff: 0.48, power:10) was applied to the reconstructed images. Reconstruction was performed with the GE Xeleris workstation using the ordered-subset expectation maximization (OSEM) algorithm with 2 iterations and 10 subsets. CT images (140 kV, 2.5 mA) with a 5 mm slice thickness and a matrix size of 512 × 512 were acquired for attenuation correction.

Two scatter correction techniques were employed for each acquisition: the commercially available, on the Xeleris workstation, DEW method [27] and the custom-made MatLab TEW algorithm, as described by Hadjiconstanti et al. [19]. The main I-123 energy window for both the DEW and TEW techniques was defined at ±10% over the 159 keV photopeak (143.1–174.9 keV). In addition, a scatter window was defined at 113 ± 10% keV (101.7–124.3 keV) for the DEW technique and two scatter windows were defined at 137 ± 1.82% keV (lower energy scatter window: 134.5–139.5 keV) and 179.9 ± 1.39% keV (upper energy scatter window: 177.4–181.4 keV) for the TEW technique [9].

Table 1 presents the volume of each remnant within the phantom, the administered activity within the corresponding remnant and the background-to-remnant activity ratio (R_bkg_) for each performed SPECT/CT acquisition. Three different sections, with two remnants in each section of specific sizes and locations, were used for these acquisitions (Figure 1). Any section could be easily attached to the phantom to study different sizes of remnants [26].

First, the response of the SPECT/CT modality to the administered I-123 activities (0.5–12 MBq) was investigated for the non-scatter-corrected (NSC) and scatter-corrected (DEW and TEW) SPECT/CT images. For this purpose, the total counts in the 1.5 and 3 mL thyroid remnants were measured. Note that these acquisitions were performed with no background activity. To avoid underestimating the response of the SPECT modality due to partial volume effects [12], large elliptical ROIs were drawn in each slice with a presented remnant using the ImageJ software package (Version 1.53) [28]. The total counts within a remnant were obtained by measuring the counts in each ROI and then summing the counts from all ROIs. 

Second, the known volumes of remnants were measured from the NSC, DEW and TEW scatter-corrected SPECT/CT and CT images to investigate the effect of SC on these measurements. Due to the small sizes of remnants and the limited spatial resolution of the SPECT modality, the cross-sectional area of a remnant in each slice was measured. The cross-sectional areas of a remnant were summed and then multiplied by the slice thickness to obtain the volume [29]. Since the cross-sectional area in each slice had an elliptical shape, it was calculated as Area = *π* α β, where α and β are the semi-major and semi-minor axes of the eclipse, respectively. Using the ImageJ software (Version 1.53), α and β were defined as the full width at half maximum (FWHM) of the line profiles across the lateral and AP directions of a slice, respectively (Figure 1). The corresponding CT slices were utilized to select the SPECT slices with remnants. Due to the high resolution of the CT modality, the cross-sectional areas and volumes from the CT images were measured using a thresholding technique and the Wand Tool in the ImageJ software.

Third, the effect of DEW and TEW scatter correction on the background region (R_bkg_ = 5% and 10%) was investigated by drawing line profiles in the SPECT/CT slices and measuring the average counts (Avg) and the corresponding noise or standard deviation (SD), and by calculating the corresponding coefficient of variation (%COV) as follows [16]:

(1) $$\%COV=\left|\frac{Noise}{Avg}\right|\times 100$$

which describes the variability or non-uniformity of the signal [30].

Next, the effect of DEW and TEW scatter correction on the image quality was investigated. For this purpose, SPECT/CT images from acquisitions with 1.5 and 3 mL were utilized to calculate the contrast-to-noise (CNR) and the signal-to-noise (SNR) ratios. The administered activity within the remnants was 0.37 MBq/mL and the R_bkg_ values were 5% and 10%. These activities represent clinically realistic scenarios. The CNR is a measure of remnant detectability in the presence of noise [31], and it was calculated as follows [16]:

(2) $$CNR=\left|\frac{\bar{C}-{\bar{C}}_{bkg}}{\sigma_{bkg}}\right|$$

where $\bar{C}$ is the average number of counts in the remnant, ${\bar{C}}_{bkg}$ is the average number of counts in the background region and $\sigma_{bkg}$ is the corresponding standard deviation. CT slices were used to select the SPECT slices with a remnant. ROIs were drawn around the remnant in all selected slices. The counts in the formed volume of interest (VOI) were measured and summed to calculate $\bar{C}$ by dividing the counts over the number of pixels within the VOI. ${\bar{C}}_{bkg}$ and $\sigma_{bkg}$ were calculated by taking the VOI in the background region. The ROIs for the corresponding $\bar{C}$ and ${\bar{C}}_{bkg}$ were selected from the same slices, and they were having the same number of pixels in each slice. The SNR for each remnant, which compares the signal of the remnant to the background signal, was calculated as follows [32]:

(3) $$SNR=\left|\frac{\bar{C}}{\sigma_{bkg}}\right|$$

Lastly, the non-scatter- and scatter-corrected SPECT/CT images with different background-to-remnant activity ratios were visually evaluated by two experienced nuclear medicine physicians.

## 3. Results

**Figure 2:**
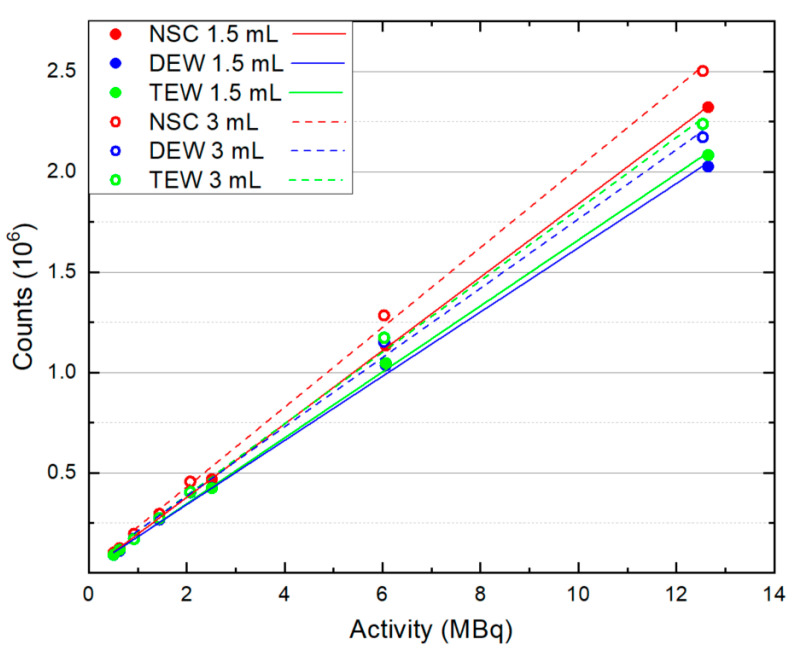
Counts with respect to administered activity within the 1.5 and 3 mL remnants from NSC, DEW and TEW scatter-corrected SPECT/CT images. A linear fit was applied to each dataset.

Figure 2 presents the total counts measured in each of the 1.5 and 3 mL remnants from NSC, DEW and TEW scatter-corrected SPECT/CT images with respect to the administered activity. A linear fit was applied to each dataset, demonstrating a linear relationship between counts and the range of administered activity for both remnant sizes, with and without scatter correction. Activities higher than 12.5 MBq were not investigated since the high count rate of I-123 did not allow a correct representation of counts within pixels. It can be observed that both scatter correction techniques reduced the number of detected photons compared to NSC images in these small remnants. On average, for the examined activities and when no background was presented, the DEW SC technique decreased the detected photons by 11.5% while the TEW one decreased them by 9.5%. More specifically, at low activities (<2.5 MBq), both SC techniques reduced the photons to a similar extent, while at higher activities (2.5–12.5 MBq), the DEW SC technique reduced photons to a higher extent.

**Figure 3:**
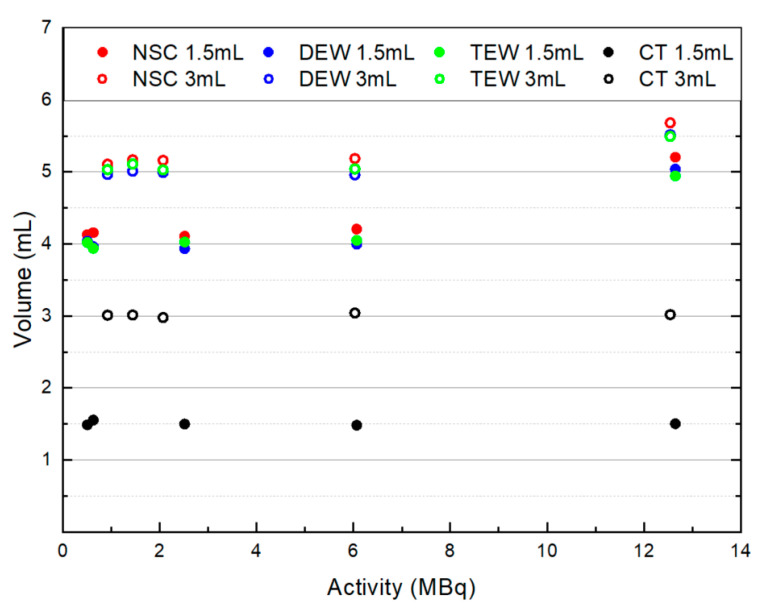
Measured volumes with respect to administered activities for the 1.5 and 3 mL remnants from the NSC, DEW and TEW scatter-corrected SPECT/CT images as well as from the CT images.

Figure 3 presents the measured volumes with respect to the administered activities from the NSC, DEW and TEW scatter-corrected SPECT/CT images as well as from the CT images. The actual volumes of remnants in the phantom were 1.5 and 3 mL. The measured volumes with and without scatter correction were overestimated due to the limited resolution of SPECT and due to partial volume effects (PVEs). The SPECT resolution with LEHR collimators is 7.4 mm at a distance of 10 cm between the collimator and source, and 11 mm at a distance of 20 cm [33]. On average, from all images, the measured volumes were 2.9 and 1.8 times higher than the known 1.5 and 3 mL remnants, respectively. The volume overestimation appeared to be slightly worsened as the administered activity was increased. Both DEW and TEW SC techniques seemed to reduce the volume overestimation to a small extent. In contrast, the measured volumes from the CT images were found to be similar to the actual ones (<1%) due to the high resolution of the CT images (1 mm). No significant differences were observed in the measured volumes from the CT images for different administered activities with and without SC.

Table 2 presents the actual volume of remnants (0.5–10 mL) and the ratio of measured over actual volume (R) from the NSC, DEW and TEW scatter-corrected SPECT/CT acquisitions of similar administered activities (2.15–3.15 MBq). The corresponding ratios from the CT images are also shown. In all images, the volume of the 10 mL remnant was accurately measured. DEW and TEW SC techniques slightly reduced the ratios. For the 0.5–1.5 mL remnants, the measured lateral and AP FWHMs in the SPECT/CT slices were 3–5 pixels, with a pixel size of 4.42 mm. Thus, these volumes could not accurately be measured. However, they could accurately be measured from the CT images since the corresponding pixel size was 1 mm.

**Figure 4:**
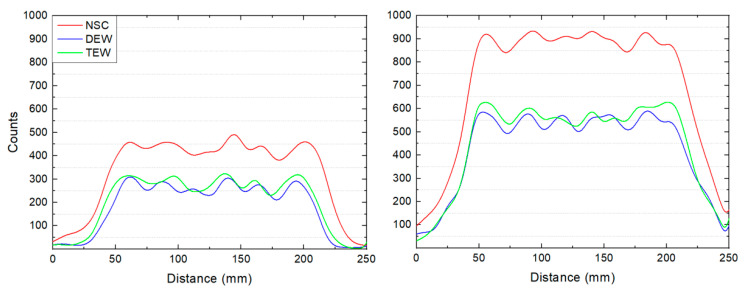
Counts from the line profiles in the lateral direction of slices with a uniform background R_bkg_ of (**left**) 5% and (**right**) 10%, located between the trachea and the remnants of the phantom, from NSC, DEW and TEW scatter-corrected SPECT/CT images.

Figure 4 shows the measured counts by drawing line profiles in the lateral direction of slices with a uniform background (R_bkg_ of 5% and 10%) from the NSC, DEW and TEW scatter-corrected SPECT/CT images. The selected slices were located between the trachea and the remnants of the phantom [26]. The average background counts, the standard deviation (noise) and the %COV from these slices are shown in Table 3. Both scatter correction techniques significantly reduced the average background counts. Particularly, at an R_bkg_ of 5%, the DEW and TEW SC techniques decreased the average background counts by 41% and 35%, respectively, while at an R_bkg_ of 10%, DEW and TEW decreased the corresponding counts by 39% and 37%, respectively, However, DEW and TEW SC decreased the noise (SD) to a smaller extent, by about 11% at an R_bkg_ of 5% and 4.5% at an R_bkg_ of 10%. Therefore, scatter correction increased the %COV. The highest increase in %COV was observed when DEW SC was applied at an R_bkg_ of 5%.

**Figure 5:**
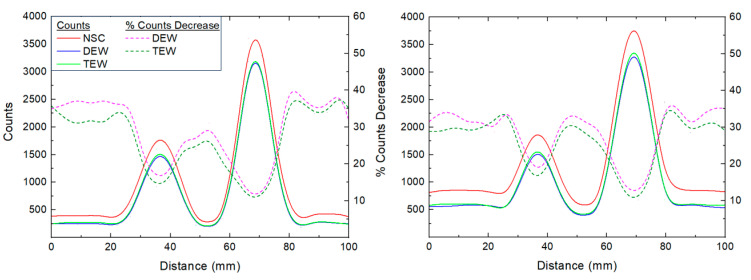
Counts from the line profiles in the lateral direction through the central slice of the 1.5 and 3 mL remnants with R_bkg_ of (**left**) 5% and (**righ**t) 10% from the NSC, DEW and TEW scatter-corrected SPECT/CT images. The administered activity within the remnants was 0.37 Bq/mL. The solid lines represent the counts from the line profile while the dashed lines represent the % decrease in counts when applying DEW and TEW SC compared to NSC images.

Figure 5 shows the measured counts by drawing line profiles in the lateral direction through the central slice of the 1.5 and 3 mL remnants at R_bkg_ values of 5% and 10% from the NSC, DEW and TEW scatter-corrected SPECT/CT images. The administered activity within the remnants was 0.37 MBq/mL. The % decrease in counts when applying each of the two SC techniques, compared to the non-scatter-corrected counts, is also shown in the figure. Both SC techniques decreased the counts in the background region, on average by about 35%. The corresponding decrease within the 1.5 and 3 mL remnants was, on average, 15% and 11%, respectively.

**Figure 6:**
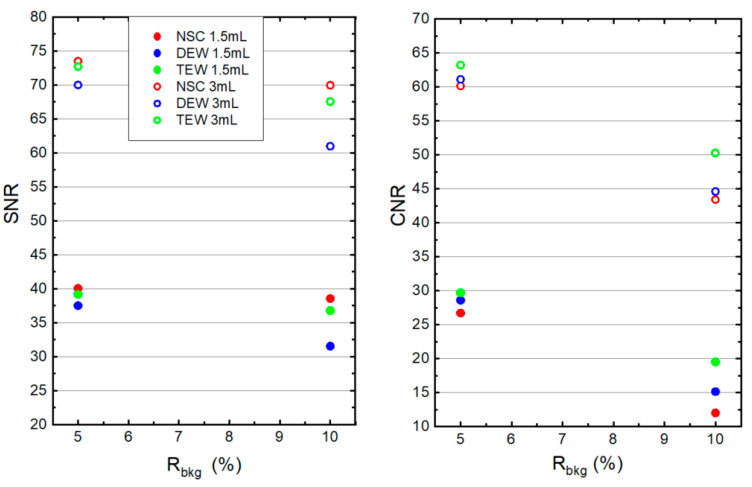
(**Left**) SNR and (**right**) CNR values for the 1.5 and 3 mL remnants measured from the NSC, DEW and TEW scatter-corrected SPECT/CT images. The administered activity within the remnants was 0.37 Bq/mL and the R_bkg_ was 5% and 10%.

Figure 6 presents the SNR and CNR values for the 1.5 and 3 mL remnants at an R_bkg_ of 5% and 10% from NSC, DEW and TEW scatter-corrected SPECT/CT images. The administered activity within the remnants was 0.37 Bq/mL. It can be observed that all SNR and CNR values are lower for the smaller-sized remnant. In addition, all SNR and CNR values are lower at R_bkg_ = 10%. Moreover, SNR values are higher for non-scattered images than the scattered-corrected images. This is because SC decreases photons and noise to almost a similar extent (Equation (3)). In all cases, the SNR and CNR values were higher when applying TEW SC compared to the DEW SC technique. Statistical errors for the SNR and CNR values were calculated using propagation of error in Equations (2) and (3). Taking into account these errors, significant differences in the SNR and the CNR between TEW and DEW SC were found for both remnants only at an R_bkg_ of 10%.

**Figure 7:**
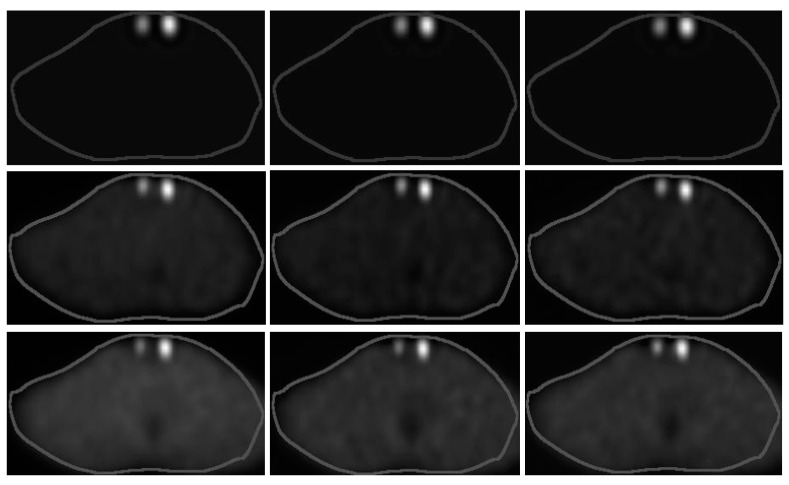
(**Left column**) NSC, (**middle column**) DEW and (**right column**) TEW scatter-corrected SPECT/CT images without (**top row**) and with an R_bkg_ of (**middle row**) 5% and (**bottom row**) 10%. Table 1 (acquisitions 1, 8, 9) presents the administered activities within the 1.5 (left side of each slice) and 3 mL (right side of each slice) remnants.

Figure 7 shows the NSC, DEW and TEW scatter-corrected SPECT/CT images without and with an R_bkg_ of 5% and 10%. Very similar activities per mL were administered within the 1.5 and 3 mL remnants. The physicians reported that they could not distinguish significant differences among the images without background activity. However, they reported that the image contrast for both remnants was improved at an R_bkg_ of 10% compared to an R_bkg_ of 5% when applying the TEW SC technique, and thus, they were more confident in assessing the volume difference between the two remnants.

## 4. Discussion

The present study investigated the impact of two scatter correction techniques (TEW and DEW SC) on I-123 SPECT/CT postsurgical thyroid imaging by using an anthropomorphic neck–thyroid phantom with small sizes of remnants surrounded by anatomically correct scattering structures. SC should be applied in this imaging since the high-energy photons emitted from I-123 penetrate the septa of the LEHR collimators and they are detected in the main I-123 photopeak window, degrading the image quality [6,9].

Window-based SC techniques are the most widely used [10,11,12]. The DEW SC technique is commercially available in most of the SPECT modalities, while TEW SC algorithms are easily accessible [19].

The response of the SPECT modality to I-123-administered activities within small sizes of remnants and with no background activity was firstly investigated. A linear relationship was exhibited for the NSC, DEW and TEW scatter-corrected SPECT/CT data between remnant uptake (counts) and administered activities from 0.5 to 12.5 MBq. In the higher range of these activities, DEW SC reduced the photons slightly more than the TEW SC technique. This could be due to the broader energy width of the scatter window used in the DEW SC technique. The chosen energy widths and centroids of the scatter windows in both SC techniques were similar to those found by Lagerburg et al. [9], where the optimal scatter windows were decided after a systematic study.

The effects of SC on volume calculation were investigated by calculating the volumes of remnants from NSC and scatter-corrected SPECT/CT images. The measured volumes of small remnants (0.5–3 mL) were overestimated. The degree of overestimation was higher for smaller remnants. This was due to the limited SPECT resolution and the PVE, as explained in the previous section. Both SC techniques slightly improved the value of the measured volume since the measured FWHMs of the line profiles were slightly decreased when applying SC. This was also reported in another study [34].

The noise and non-uniformity in background regions (R_bkg_ = 5% and 10% of 0.37 MBq/mL) were also investigated for NSC and scatter-corrected SPECT/CT images. SC significantly reduced photons. DEW SC decreased photons more than the TEW SC technique due to the broader energy width of its scatter window, and in particular, to a higher extent at an R_bkg_ of 10%. This scatter compensation with the increase in the background activity was also reported in an I-131 study [35].

The effect of SC depends on several factors such as the background activity, the object’s size and shape, and the scattering medium or surrounding structures [35]. In this study, the degree of photon decrease was also higher in larger regions (background) than in smaller regions (remnants).

The extent that SC decreased the counts within the remnants as well as the standard deviation of the background counts did not increase the SNR values when applying SC compared to the corresponding NSC ones. This was also found in another Tc-99m phantom study with different sizes of lesions [36]. As expected, the SNR and CNR values were higher for the larger remnant and for the smaller R_bkg_. Moreover, TEW SC, compared to the DEW SC technique, resulted in higher SNR and CNR values. However, these differences were significant only for the higher R_bkg_. In addition, improved image contrasts were reported by the physicians for the 1.5 and 3 mL remnants when TEW SC was applied at an R_bkg_ of 10% compared to DEW SC.

Different energy widths and centroids for the scatter windows of these SC techniques may slightly change the photon decrease. However, the subject of this study was to investigate the impact of DEW and TEW SC on I-123 SPECT/CT images in relation to the measured FWHMs and volumes, and the photon decreases in the background and remnant regions for different administered activities. It is important to study this effect using clinically realistic activities and anthropomorphic phantoms with tissue-equivalent, human-shaped and anatomically correct surrounding structures. Specifically, for postsurgical thyroid imaging scenarios, the examined remnants should be in clinically relevant positions within proper anthropomorphic phantoms. Thus, the effect of scattering on the smallest detectable remnants can be studied.

A combined approach of ultrasound (US) and SPECT imaging can offer an early and comprehensive assessment of the risk of persistent or recurrent disease according to the European Thyroid Association [37]. US is a non-irradiating imaging technique that can detect structural abnormalities, whereas SPECT is more sensitive in detecting small or functional changes (e.g., small amounts of residual thyroid tissue or cancer cells). In simple cases, US can prevent the use of other types of imaging, and in complex cases, it can enable fast consideration of other types of imaging [38]. The timing and frequency for performing each type of imaging are typically determined on a case-by-case basis by the involved healthcare professionals following the guidelines.

The application of 3D printing technology for individualized applicators and the substantial impact of image-guided adaptive external beam radiotherapy and brachytherapy on personalized medicine were emphasized in a review study [39]. SPECT/CT imaging, as well as dedicated or individualized phantoms, can also be utilized for these radiological procedures.

In the future, other SC techniques will be investigated using the same phantom, and they will be quantitatively and qualitatively compared with the DEW and TEW SC techniques.

## 5. Conclusions

The impact of DEW and TEW scatter correction on I-123 SPECT/CT imaging was quantitatively and qualitatively investigated using an anthropomorphic phantom with small sizes of remnants in clinically relevant positions and surrounded by tissue-equivalent and anatomically correct scattering structures.

Scatter correction slightly improves the volume calculation from SPECT/CT images. Volumes of small remnants should be measured from CT images due to PVEs and the limited SPECT resolution. Scatter correction decreases photons to a higher extent in larger regions than smaller regions. Larger sizes of remnants present higher SNR and CNR values, particularly at lower background activities. From the quantitative analysis and the qualitative evaluation, TEW scatter correction is preferable over DEW scatter correction, particularly at higher background activities, while no significant differences can be observed between them at lower background activities.

Scatter correction should be applied in I-123 postsurgical SPECT/CT imaging to improve the image contrast and detectability of small volumes.

## Figures and Tables

**Table 1:** The volume (V_i_) of each remnant within the phantom, the administered activity within the corresponding remnant (A_i_) and the background-to-remnant activity ratio (R_bkg_) for each acquisition (ID).

ID	V_1_ (mL)	A_1_ (MBq)	V_2_ (mL)	A_2_ (MBq)	R_bkg_ (%)
1	1.5	0.51	3.0	0.93	0
2	1.5	0.63	3.0	1.44	0
3	1.5	2.52	3.0	2.07	0
4	1.5	6.07	3.0	6.03	0
5	1.5	12.65	3.0	12.54	0
6	10.0	3.15	0.5	2.15	0
7	10.0	3.15	1.0	2.52	0
8	1.5	0.55	3.0	1.11	5
9	1.5	0.55	3.0	1.11	10

**Table 2:** The actual volume (V) of remnants and the ratio of measured over actual volume (R) from the NSC, DEW and TEW scatter-corrected SPECT/CT images and CT images with similar administered activities (A in MBq) shown in Table 1.

V (mL)	R_NSC_	R_DEW_	R_TEW_	R_CT_
0.5	5.8	5.5	5.6	1.1
1.0	2.9	2.7	2.6	1.1
1.5 *	2.8	2.6	2.5	1.0
3.0 *	1.8	1.6	1.6	1.0
10.0	1.1	1.1	1.1	1.0

* ID = 3 from Table 1.

**Table 3:** The average background counts (Avg), the standard deviation or noise (SD) and the %COV from the line profiles in Figure 4.

R_bkg_ = 5%				R_bkg_ = 10%		
	Avg	SD	%COV	Avg	SD	%COV
NSC	441.2	27.6	6.2	895.3	31.4	3.5
DEW	262.2	25.2	9.6	544.6	30.2	5.5
TEW	288.7	24.0	8.3	566.3	29.8	5.3

## Data Availability

The data presented in this study are available upon request from the corresponding authors.

## Abbreviations

3D	Three-dimensional
A	Activity
AP	Anterior-Posterior
Avg	Average
Bgk	Background
CNR	Contrast-to-Noise Ratio
COV	Coefficient of Variation
CT	Computed Tomography
DEW	Dual Energy Window
FWHM	Full Width at Half Maximum
LEHR	Low-Energy High-Resolution
MIBG	Meta-Iodo-Benzyl-Guanidine
NSC	Non-Scatter Corrected
PVE	Partial Volume Effect
Rbkg	Background-to-Remnant Activity Ratio
ROI	Region of Interest
SC	Scatter Correction
SD	Standard Deviation
SNR	Signal-to-Noise Ratio
SPECT	Single-Photon Emission Computed Tomography
TEW	Triple Energy Window
US	Ultrasound
V	Volume
VOI	Volume of Interest

## References

[1] Siddiqi A., Foley R.R., Britton K.E., Sibtain A., Plowman P.N., Grossman A.B., Monson J.P., Besser G.M. (2001). The Role of 123I-Diagnostic Imaging in the Follow-up of Patients with Differentiated Thyroid Carcinoma as Compared to 131I-Scanning: Avoidance of Negative Therapeutic Uptake due to Stunning. Clin. Endocrinol..

[2] Mandel S.J., Shankar L.K., Benard F., Yamamoto A., Alavi A. (2001). Superiority of Iodine-123 Compared with Iodine-131 Scanning for Thyroid Remnants in Patients with Differentiated Thyroid Cancer. Clin. Nucl. Med..

[3] Barwick T., Murray I., Megadmi H., Drake W.M., Plowman P.N., Akker S.A., Chew S.L., Grossman A.B., Avril N. (2010). Single Photon Emission Computed Tomography (SPECT)/Computed Tomography Using Iodine-123 in Patients with Differentiated Thyroid Cancer: Additional Value over Whole Body Planar Imaging and SPECT. Eur. J. Endocrinol..

[4] Cascini G.L., Niccoli Asabella A., Notaristefano A., Restuccia A., Ferrari C., Rubini D., Altini C., Rubini G. (2014). ^124^Iodine: A Longer-Life Positron Emitter Isotope—New Opportunities in Molecular Imaging. BioMed Res. Int..

[5] Gunder D.L., Wernick M.N., Aarsvold J.N. (2004). Collimator Design for Nuclear Medicine. Emission Tomography: The Fundamentals of PET and SPECT.

[6] Small A.D., Prosser J., Motherwell D.W., McCurrach G.M., Fletcher A.M., Martin W. (2006). Downscatter Correction and Choice of Collimator in ^123^I Imaging. Phys. Med. Biol..

[7] Zaidi H., Hasegawa B.H., Zaidi H. (2006). Attenuation Correction Strategies in Emission Tomography. Quantitative Analysis in Nuclear Medicine Imaging.

[8] Chen J. (2021). Nuclear Data Sheets for A = 123. Nucl. Data Sheets.

[9] Lagerburg V., de Nijs R., Holm S., Svarer C. (2012). A Comparison of Different Energy Window Subtraction Methods to Correct for Scatter and Downscatter in I-123 SPECT Imaging. Nucl. Med. Commun..

[10] Hutton B.F., Buvat I., Beekman F.J. (2011). Review and Current Status of SPECT Scatter Correction. Phys. Med. Biol..

[11] Ljungberg M., Pretorius P.H. (2018). SPECT/CT: An Update on Technological Developments and Clinical Applications. Br. J. Radiol..

[12] Ritt P., Kuwert T., Schober O., Kiessling F., Debus J. (2020). Quantitative SPECT/CT—Technique and Clinical Applications. Molecular Imaging in Oncology.

[13] Noori-Asl M., Sadremomtaz A., Bitarafan-Rajabi A. (2014). Evaluation of Three Scatter Correction Methods Based on Estimation of Photopeak Scatter Spectrum in SPECT Imaging: A Simulation Study. Phys. Medica.

[14] Farncombe T.H., Gifford H.C., Narayanan M.V., Pretorius P.H., Frey E.C., King M.A. (2004). Assessment of Scatter Compensation Strategies for 67Ga SPECT Using Numerical Observers and Human LROC Studies. J. Nucl. Med..

[15] Pourmoghaddas A., Vanderwerf K., Ruddy T.D., Glenn Wells R. (2015). Scatter Correction Improves Concordance in SPECT MPI with a Dedicated Cardiac SPECT Solid-State Camera. J. Nucl. Cardiol..

[16] Van Gils C.A.J., Beijst C., Van Rooij R., De Jong H.W.A.M. (2016). Impact of Reconstruction Parameters on Quantitative I-131 SPECT. Phys. Med. Biol..

[17] Mu’minah I.A.S., Hidayati N.R., Widodo P., Shintawati R., Soejoko D.S. (2020). Investigation of Image Quality for Quantitative Lu-177 in SPECT Imaging: A Phantom Study. J. Phys. Conf. Ser..

[18] Miwa K., Nemoto R., Masuko H., Yamao T., Kobayashi R., Miyaji N., Inoue K., Onodera H. (2022). Evaluation of Quantitative Accuracy among Different Scatter Corrections for Quantitative Bone SPECT/CT Imaging. PLoS ONE.

[19] Hadjiconstanti A., Michael K., Frangos S., Demosthenous G., Lyra M. The Impact of Two Scatter Correction Methods on I-131 AC-SPECT Images Using an Anthropomorphic Phantom with Variable Sizes of Thyroid Remnants. Proceedings of the 2020 7th International Conference on Biomedical and Bioinformatics Engineering.

[20] Fletcher A.M., Motherwell D.W., Small A.D., McCurrach G.M., Goodfield N.E.R., Petrie M.C., Martin W., Cobbe S.M. (2010). I-123 MIBG Cardiac Uptake Measurements: Limitations of Collimator Choice and Scatter Correction in the Clinical Context. Nucl. Med. Commun..

[21] Kobayashi H., Momose M., Kanaya S., Kondo C., Kusakabe K., Mitsuhashi N. (2003). Scatter Correction by Two-Window Method Standardizes Cardiac I-123 MIBG Uptake in Various Gamma Camera Systems. Ann. Nucl. Med..

[22] Inoue Y., Shirouzu I., Machida T., Yoshizawa Y., Akita F., Minami M., Ohtomo K. (2004). Collimator Choice in Cardiac SPECT with I-123-Labeled Tracers. J. Nucl. Cardiol..

[23] Papanastasiou E., Moralidis E., Siountas A. (2017). The Effect of Scatter Correction on Planar and Tomographic Semiquantitative I123 Cardiac Imaging. A Phantom Study. Hell. J. Nucl. Med..

[24] Hayashi M., Deguchi J., Utsunomiya K., Yamada M., Komori T., Takeuchi M., Kanna K., Narabayashi I. (2005). Comparison of Methods of Attenuation and Scatter Correction in Brain Perfusion SPECT. J. Nucl. Med. Technol..

[25] Yang Y.-W., Chen J.-C., Chang C.-J., Cheng C.-Y., Wang S.-J. (2008). Evaluation of Collimator Choice and Scatter Correction on 123I SPECT Images. Nucl. Instrum. Methods Phys. Res. Sect. A Accel. Spectrometers Detect. Assoc. Equip..

[26] Michael K., Hadjiconstanti A., Lontos A., Demosthenous G., Frangos S., Parpottas Y. (2023). A Neck-Thyroid Phantom with Small Sizes of Thyroid Remnants for Postsurgical I-123 and I-131 SPECT/CT Imaging. Life.

[27] Koral K.F., Swailem F.M., Buchbinder S., Clinthorne N.H., Rogers W.L., Tsui M.W. (1990). SPECT Dual-Energy-Window Compton Correction: Scatter Multiplier Required for Quantification. J. Nucl. Med..

[28] Rueden C.T., Schindelin J., Hiner M.C., DeZonia B.E., Walter A.E., Arena E.T., Eliceiri K.W. (2017). ImageJ2: ImageJ for the next Generation of Scientific Image Data. BMC Bioinform..

[29] Brenner D.E., Whitley N.O., Houk T.L., Aisner J., Wiernik P., Whitley J. (1982). Volume Determinations in Computed Tomography. JAMA J. Am. Med. Assoc..

[30] BIPM, IEC, IFCC, ILAC, ISO, IUPAC, IUPAP, OIML (2008). Evaluation of Measurement Data-Guide to the Expression of Uncertainty in Measurement. JCGM 100:2008 (GUM 1995 with Minor Corrections).

[31] Cherry S., Sorenson J., Phelps M. (2012). Physics in Nuclear Medicine.

[32] Wieczorek H. (2006). SPECT Image Quality and Quantification. Proceedings of the IEEE Nuclear Science Symposium Conference Record.

[33] Tunninen V., Kauppinen T., Eskola H., Eskola H., Väisänen O., Viik J., Hyttinen J. (2018). Physical Characteristics of Collimators for Dual-Isotope Imaging with 99mTc and 123I. EMBEC & NBC 2017.

[34] Fakhri G.E., Benali H., Todd-Pokropek A., Paola R.D. (2000). Relative Impact of Scatter, Collimator Response, Attenuation, and Finite Spatial Resolution Corrections in Cardiac SPECT. J. Nucl. Med..

[35] Dewaraja Y.K., Ljungberg M., Koral K.F. (2000). Accuracy of 131I Tumor Quantification in Radioimmunotherapy Using SPECT Imaging with an Ultra-High-Energy Collimator: Monte Carlo Study. J. Nucl. Med..

[36] Perisinakis K., Karkavitsas N., Damilakis J., Gourtsoyiannis N. (1998). Effect of Dual and Triple Energy Window Scatter Correction Methods on Image Quality in Liver Scintigraphy. Nuklearmedizin.

[37] Leenhardt L., Erdogan M.F., Hegedus L., Mandel S.J., Paschke R., Rago T., Russ G. (2013). 2013 European Thyroid Association Guidelines for Cervical Ultrasound Scan and Ultrasound-Guided Techniques in the Postoperative Management of Patients with Thyroid Cancer. Eur. Thyroid J..

[38] Vrinceanu D., Dumitru M., Cergan R., Anghel A.G., Costache A., Patrascu E.T., Sarafoleanu C.C. (2018). Correlations between Ultrasonography Performed by the ENT Specialist and Pathologic Findings in the Management of Three Cases with Thyroglossal Duct Cyst. Med. Ultrason..

[39] Anghel B., Serboiu C., Marinescu A., Taciuc I.-A., Bobirca F., Stanescu A.D. (2023). Recent Advances and Adaptive Strategies in Image Guidance for Cervical Cancer Radiotherapy. Medicina.

